# Shedding light on photomorphogenesis

**DOI:** 10.1093/plcell/koae218

**Published:** 2024-07-23

**Authors:** Vicky Howe

**Affiliations:** Assistant Features Editor, The Plant Cell, American Society of Plant Biologists; Department of Developmental Genetics, Heinrich-Heine University, Düsseldorf, Germany, 40225

Light is important for plants, not only because it powers photosynthesis but also because it serves as an environmental cue, indicating the seasons based on the day length or the plant's position, be it in sun or shade. Plants growing in full sunlight are exposed to roughly equal ratios of red and far-red light. However, those in another plant's shadow receive a higher proportion of far-red light, as the shorter wavelengths are absorbed by the other plant's leaves, which hinders photosynthesis. Plants therefore need to be able to perceive the quantity and quality of light they receive so they can adjust their growth and development accordingly, such as by flowering at the correct time of year or by avoiding the shade of another plant by elongating and growing towards the light. One way they do this is through the phytochrome (phy) family of photoreceptors (comprising phyA–E), which perceive red and far-red (∼650–730 nm) light.

Phys are important regulators of light-dependent growth or photomorphogenesis. They are also necessary for the shade avoidance response. When exposed to red light, phy proteins are converted from their inactive, cytoplasm-localized, red-absorbing Pr form to the active far-red–absorbing Pfr. Pfr rapidly reverts to inactive Pr upon exposure to far-red light, eliciting shade avoidance responses but also gradually in darkness in a process called thermal reversion (Klose et al. 2020). The ratio of Pr to Pfr at daybreak can therefore serve as an indicator of day length to regulate season-dependent plant growth (Hendricks 1960).

Unlike Pr, activated Pfr can move to the nucleus, where it interacts with transcription factors of the PHYTOCHROME-INTERACTING FACTOR (PIF) family. In darkness, such as when seeds are buried within the soil, PIFs act as repressors of photomorphogenesis, favoring skotomorphogenesis, or dark-dependent growth, which includes hypocotyl elongation as the plant grows toward the surface. Once the seedling reaches the surface and there is sufficient light, red light–activated Pfr induces phosphorylation, ubiquitination, and subsequent proteasomal degradation of PIFs, allowing the upregulation of light-dependent genes to promote photomorphogenesis (Cai and Huq 2024) (see Fig.). While a lot of research has been done on PIF degradation, relatively little is known about PIF stabilization via dephosphorylation. This latest study by **Xingbo Cai and coauthors (Cai et al. 2024)** sheds some light on this matter.

**Figure.**
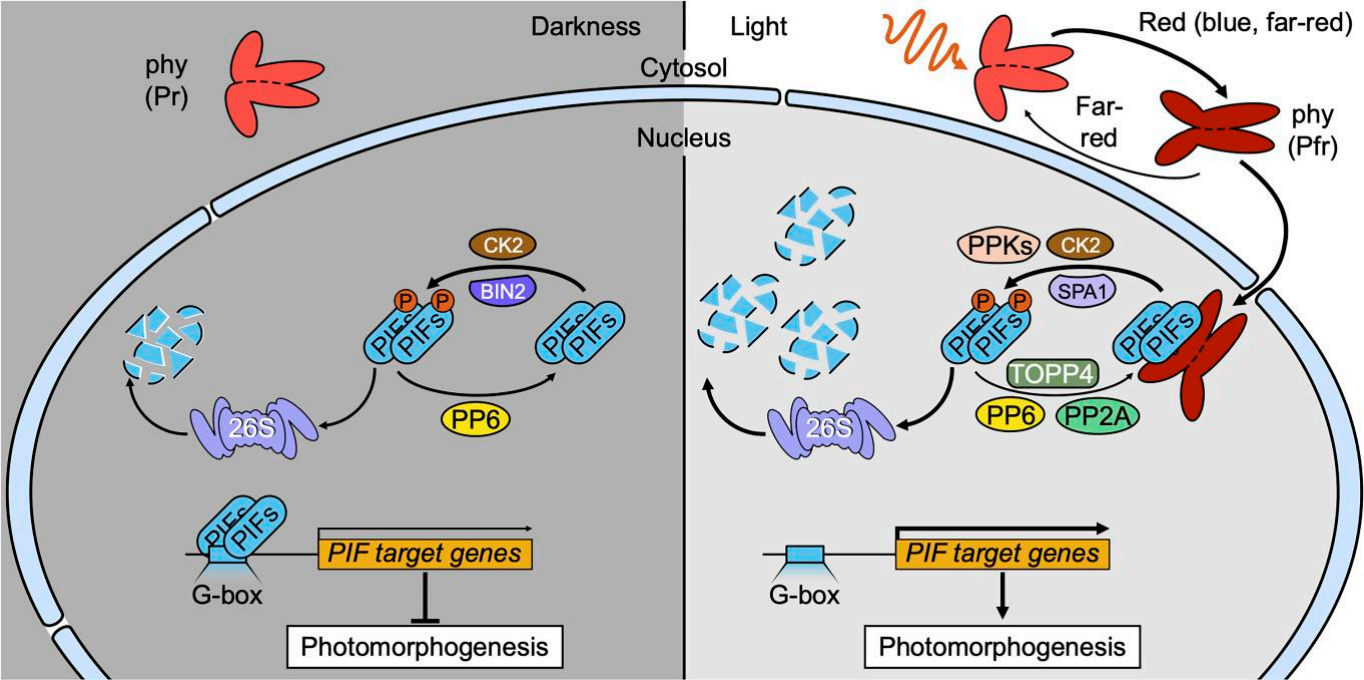
The phy light-signaling pathway regulates photomorphogenesis via phosphorylation and dephosphorylation of PIFs. Pr is inactive in darkness and remains in the cytosol. Upon light exposure, red light converts Pr to active Pfr, which can move to the nucleus and induce phosphorylation of PIFs via kinases such as PPKs, CK2, and SPA1, leading to their proteasomal degradation to promote photomorphogenesis. This phosphorylation can be reversed by phosphatases, including PP2A, TOPP4, and PP6, which stabilize PIFs and allow them to inhibit photomorphogenesis. Figure from Cai et al. (2024), Figure 8.

To identify phosphatases that might dephosphorylate PIFs, the group used PIF3 as a bait in a yeast 2-hybrid screen. PIF3 is the founding member of the PIF family and facilitates hypocotyl elongation in red light by inhibiting phyB signaling (Kim et al. 2003). Four different isoforms of the regulatory “B” subunit of PROTEIN PHOSPHATASE2A (PP2A) were found to interact with PIF3. To determine whether these PP2A B subunits regulate phyB-dependent photomorphogenesis, Cai and colleagues generated various combinations of PP2A B subunit knockout and overexpression lines in Arabidopsis and examined the hypocotyl length of seedlings grown under dark or red-light conditions.

As it transpired, the 4 PP2A B subunits function redundantly, and in the same genetic pathway, together with PIF3 to promote hypocotyl elongation in red light. Furthermore, when exposed to red light, PIF3 was degraded faster and had higher levels of phosphorylation in *pp2a b* mutants than in wild-type plants, suggesting the PP2A B subunits inhibit red light–induced degradation of PIF3 by promoting its dephosphorylation. However, the authors are careful to note that PP2A is unlikely to be the only phosphatase targeting PIF3 and that it may only act on a subset of phosphorylation sites, as several kinases are already known to phosphorylate PIFs in both dark and light conditions (Fig.).

Thus, this study has revealed another level of negative regulation to fine-tune photomorphogenesis via light-dependent dephosphorylation and stabilization of PIF3, adding to the accumulating knowledge of the intricate mechanisms that plants use to perceive and react to their environment.
